# Microchannel Gas Flow in the Multi-Flow Regime Based on the Lattice Boltzmann Method

**DOI:** 10.3390/e26010084

**Published:** 2024-01-18

**Authors:** Xiaoyu Li, Zhi Ning, Ming Lü

**Affiliations:** School of Mechanical, Electronic and Control Engineering, Beijing Jiaotong University, Beijing 100044, China; 20116039@bjtu.edu.cn (X.L.); zhining@bjtu.edu.cn (Z.N.)

**Keywords:** lattice Boltzmann method, slip boundary condition, sensitivity analysis, multi-flow regime

## Abstract

In this work, a lattice Boltzmann method (LBM) for studying microchannel gas flow is developed in the multi-flow regime. In the LBM, by comparing previous studies’ results on effective viscosity in multi-flow regimes, the values of the rarefaction factor applicable to multi-flow regions were determined, and the relationship between relaxation time and *Kn* number with the rarefaction factor is given. The *Kn* number is introduced into the second-order slip boundary condition together with the combined bounce-back/specular-reflection (CBBSR) scheme to capture the gas flow in the multi-flow regime. Sensitivity analysis of the dimensionless flow rate to adjustable parameters using the Taguchi method was carried out, and the values of adjustable parameters were determined based on the results of the sensitivity analysis. The results show that the dimensionless flow rate is more sensitive to *j* than *h*. Numerical simulations of Poiseuille flow and pulsating flow in a microchannel with second-order slip boundary conditions are carried out to validate the method. The results show that the velocity profile and dimensionless flow rate simulated by the present numerical simulation method in this work are found in the multi-flow regime, and the phenomenon of annular velocity profile in the microchannel is reflected in the phases.

## 1. Introduction

Over the last few decades, microchannel gas flow research has greatly promoted scientific and technological progress such as the micro-electro-mechanical system (MEMS), pharmaceuticals, chemistry, semiconductor materials, biology, microfluidics, and aerospace [1,2,3,4,5,6,7,8]. Microchannel gas flow can be studied with experimental and numerical simulation methods. Due to the microchannel width from micrometers to nanometers, it is generally difficult to use experimental methods. The numerical simulation method is an alternative and cost-effective way.

In the numerical simulation method, the characteristic length of the microchannel is usually on the same order of magnitude as the gas mean free path. Therefore, the microchannel gas flow can be expressed by the Knudsen number (*Kn*), *Kn* = *λ*/*H*, where *λ* is the gas mean free path and *H* is the characteristic length of the flow domain. A well-accepted classification of gas flow by *Kn* as follows [3,9,10] the continuum flow regime (*Kn* ≤ 0.001), slip flow regime (0.001 < *Kn* ≤ 0.1), transition flow regime (0.1 < *Kn* ≤ 10), and free molecular flow regime (*Kn* > 10). In the continuum flow regime, the continuum hypothesis holds, and the gas flow satisfies the Navier–Stokes equation. In the slip flow regime, the continuum hypothesis is basically valid, and the Navier–Stokes equation, which considers slip boundary condition, is regarded as a feasible method [11,12,13,14,15]. In the transition flow, it is accepted that the conventional Navier–Stokes equations are invalid because the continuum and thermodynamic equilibrium assumptions begin to break down and the rarefaction effects dominate the flow [3,16,17]. Theoretically, microchannel gas flow in the transition flow regime can be numerically simulated by the Direct Simulation Monte Carlo (DSMC) method [18]. In the DSMC method, the number of particles distributed in the field is directly related to the number of molecules. It usually suffers from statistical noise and very expensive computational costs [17,19,20,21]. In the free molecular flow regime, the Molecular Dynamics (MD) method is usually used [22]. However, MD cannot reach scales beyond a few tens of nanometers, and the coupling between MD and fluid models must necessarily proceed through a huge gap in space and time scales [22,23,24].

The above numerical simulation methods have their limitations in multi-flow regimes. Based on the Boltzmann equation, the mesoscopic lattice Boltzmann method (LBM) has been created. The LBM has a strong physical basis, and it simulates the flow of gases by imitating the basic behavior of a gas. Molecules move forward and are scattered as they collide with one another. Therefore, it is well-accepted that the LBM can be used for gas flow in multi-flow regimes [25,26,27,28,29].

In the beginning, the standard LBM cannot simulate the microchannel gas flow in the transition flow regime. The failure can be attributed to its insufficient capability for capturing the Knudsen layer (KL) and the kinetic boundary layer near solid surfaces [20,28], or in other words, it is necessary to determine suitable relaxation times and boundary conditions in the LBM for transition flow regime.

The relaxation time is the key to capturing KL, as it determines the gas–solid interaction [28]. Since the relaxation time and the effective viscosity are interrelated [30], some expressions of effective viscosity have been proposed [13,31,32,33,34]. For example, Lilley and Sader [35] investigated gas flow in the Knudsen layer for a small *Kn* value and presented an approximation, *μ*_0_(*y*) = *μ*_0_*y*^1 − *δ*^/*Cδ*, where *μ*_0_ is the bulk dynamic viscosity, *δ* and *C* are the parameters, and *y* is the distance from the solid surface. Guo et al. [28] presented an equation between two parallel plates and considered wall distance, *μ_e_* = *μ*_0_[*Ψ*(*y*/*λ*) + *Ψ*((*H* − *y*)/*λ*)]/2, where *y* is the distance from the wall. Beskok and Karniadakis [13] proposed another correction form *μ_e_* = *μ*_0_/(1 + *rKn*), which suggested a Knudsen dependence of the rarefaction factor *r,* and Vasilis et al. [20] confirmed this expression with the DSMC method. The results show that the viscosity is not constant with *Kn* and changes by *r* within the transition regime. In the previous studies, the value of the factor *r* is not yet accurate, and the relationship between relaxation time and *Kn* has not been clear.

The boundary condition is the key to accurately capturing wall slip phenomena for rarefied gas flow. At present, Succi [24] proposed a combined bounce-back/specular-reflection (CBBSR) scheme. Tang et al. [36] proposed the Discrete Maxwellian (DM) scheme. Guo et al. [28] proposed a generalized CBBSR model for the MRT-LBE. Some independent studies have proposed the high-accuracy generalized second-order slip boundary condition with the LBM [17,19,28,37]. But, in previous studies, the problem of multi-flow regime boundary conditions has not been solved.

In this work, we intend to study microchannel gas flows in a multi-flow regime based on the LBM. According to previous studies, relaxation time and an appropriate slip boundary condition play important roles in the LBM. The rest of this paper is organized as follows. The LBM is given in Section 2, including the lattice Boltzmann equation, the relationship between relaxation time and *Kn*, and the boundary condition, considering *Kn* and key parameters. The numerical results are given in Section 3, including sensitivity analysis, Poiseuille flow, and pulsating flow in the microchannel. The conclusions are given in Section 4.

## 2. Multi-Flow Regime Based on the LBM

### 2.1. Basic Lattice Boltzmann Equation

The Boltzmann equation is derived from the kinetic theory of gases [25], in which the particle distribution function is the most fundamental. The particle distribution function includes space position **x**, time *t*, and discrete particle velocity **ξ***_i_*. The discrete Boltzmann equation is expressed as:

(1) $$f_{i}\left(x+\xi_{i}\Delta t,t+\Delta t\right)-f_{i}\left(x,t\right)=\Omega_{i}\left(f\right)-\Delta tF_{i}$$

where **F***_i_* is the forcing term, **Ω***_i_*(**x**,*t*) is the discrete collision operator, and Δ*t* is the step size in time. The discrete collision operator can be expressed by the BGK model as [26]:

(2) $$\Omega_{i}\left(f\right)={-1/\tau (f}_{i}-f_{i}^{eq})$$

where *f_i_^eq^* is the discrete equilibrium particle distribution function and *τ* is the relaxation time. The D2Q9 discrete velocity model [19] is mostly two-dimensional, and it is expressed as:

(3) $$\xi_{i}=\Delta x/\Delta t\left[\begin{array}{ccccccccc} 0 & 1 & 0 & -1 & 0 & 1 & -1 & -1 & 1\\ 0 & 0 & 1 & 0 & -1 & 1 & 1 & -1 & -1 \end{array}\right]$$

(4) $$w_{i}=\left\{\begin{array}{c} 4/9\\ 1/9\\ 1/36 \end{array}\right.\begin{array}{l} i=0\\ i=1,2,3,4\\ i=5,6,7,8 \end{array}$$

where Δ*x* is the lattice spacing and *w_i_* is the weight. In the LBM, the equilibrium distribution function is given by [19]:

(5) $$f^{eq}\left(x+\xi_{i},t\right)=\rho w_{i}\left[1+3\left(\xi_{i}\left(x,t\right)\cdot u\left(x,t\right)\right)+9\left(\xi_{i}\left(x,t\right)\cdot u\left(x,t\right)\right)/2-3u^{2}/2\right]$$

where $\rho =\sum_{i}f_{i}$, and **u** is the gas velocity. The momentum is expressed as:

(6) $$\rho u=\sum_{i}f_{i}\xi_{i}+\Delta tF/2$$

The discrete force is given by [28]:

(7) $$F_{i}=w_{i}\left(1-1/2\tau \right)\left[3\left(F\cdot \xi_{i}\right)+9\left(uF+Fu\right):(\xi_{i}\xi_{i}-1/3I)/2\right]$$

where I is the unity matrix.

### 2.2. The Relationship between Relaxation Time and Kn

Relaxation time primarily influences how quickly gas particles transition from collision to equilibrium. *Kn* represents the rarefaction degree of gas particles, which affects the collision to equilibrium process, and *Kn* inevitably also affects the relaxation time. According to the kinetic theory of gases, the relationship between gas dynamic viscosity and the gas mean free path can be expressed as [21]:

(8) $$\lambda =\mu \sqrt{\pi RT/2}/\rho$$

where *λ* is the gas mean free path, *μ* is gas dynamic viscosity, and *R* is the gas constant.

In the LBM, the relationship between gas dynamic viscosity and relaxation time can be expressed as:

(9) $$\mu =\rho RT\left(\tau -1/2\right)∆t$$

Combining Equations (8) and (9), the relaxation time and the gas mean free path can be expressed as:

(10) $$\tau =1/2+\lambda \sqrt{2/\pi RT}/∆t$$

In the LBM, the relationship between the lattice spacing and the step size in time can be expressed as:

(11) $$∆x/∆t=\sqrt{\chi RT}$$

where *χ* is the discrete model constant, whose value mainly depends on the discrete velocity model. Since the discrete velocity model used in this article is D2Q9, the discrete model constant value is 3 [38].

The *Kn* can be expressed as:

(12) Kn=λ/H

where *H* is the characteristic length. Substitute Equations (11) and (12) into Equation (10) to obtain the general relationship between relaxation time and *Kn*, which can be expressed as:

(13) $$\tau =KnH\sqrt{6/\pi }/∆x+1/2$$

The KL thickness is related to *Kn*. For the continuous flow regime and slip flow regime with *Kn* < 0.1, the velocity distribution can be predicted by Equation (13). However, in the transition flow regime with 0.1 < *Kn* ≤ 10, the KL thickness is relatively thick, and the collision frequency between gas particles in this regime is significantly reduced. The velocity distribution cannot be accurately expressed by Equation (13).

To accurately predict the velocity distribution in the continuous flow regime, slip flow regime, and transition flow regime, it is necessary to reestablish the relationship between the gas dynamic viscosity and *Kn*, which reflects the influence of the flow regime on the gas dynamic viscosity. In order to achieve the above purpose, the gas dynamic viscosity is modified by introducing a correction function. The correction function with *Kn* as the variable is also called the Bosanquet-type correction function [13,20]. The Bosanquet-type correction function can be expressed as:

(14) $$\mu_{e}=\mu /\left(1+rKn\right)$$

where *r* is the rarefaction factor and *μ_e_* is the corrected gas dynamic viscosity, i.e., the effective dynamic viscosity.

**Figure 1:**
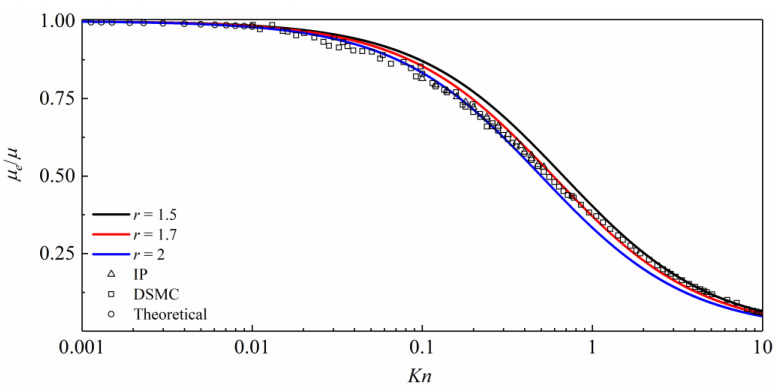
Dimensionless effective viscosity with *Kn*. The black line, the red line and the blue line are rarefied gas coefficient of 1.5 and 1.7 and 2, respectively. Triangle is the IP method by Roohi and Darbbandi [39], square is the DSMC method by Michalis et al. [20], and circle is the theoretical method by Stops [30].

The variation of dimensionless gas dynamic viscosity with *Kn* is shown in Figure 1, and the rarefied gas coefficient is taken as 2, 1.7, and 1.5. The Direct Simulation Monte Carlo (DSMC) method is mainly used in the transitional flow regime and can also be used in the slip flow regime [20], the Information Preservation (IP) method is mainly used in the transitional flow regime [39], and the theoretical methods based on the molecular free path is mainly used in the continuous flow regime and slip flow regime [30]; the above three methods are compared in Figure 1.

As can be seen in Figure 1, *Kn* ≥ 2, and the dimensionless gas effective viscosity is very close to the DSMC when the rarefaction factor is 1.5. For 0.3 < *Kn* ≤ 2, the dimensionless gas effective viscosity is very close to the DSMC and the IP when the rarefaction factor is 1.7. For 0.01 < *Kn* ≤ 0.3, the dimensionless gas effective viscosity is very close to the theoretical method, the DSMC method, and the IP method when the rarefaction factor is 2. For *Kn* ≤ 0.01, due to the macroscopic flow of the gas, the influence of the continuous flow regime on the rarefaction factor can no longer be considered.

Based on the above comparison and analysis, when simulating the multi-flow regime with the LBM, the value of the rarefaction factor in Equation (14) is taken as follows:

(15) $$r=\left\{\begin{array}{c} 2\\ 1.7\\ 1.5 \end{array}\right.\begin{array}{c} 0.001<Kn\leq 0.3\\ 0.3<Kn\leq 2\\ 2<Kn\leq 10 \end{array}$$

Substituting Equation (14) into Equation (9) and combining with Equations (8), (11), and (12), the relationship between relaxation time and *Kn* can be finally expressed as:

(16) $$\tau =KnH\sqrt{6/\pi }/∆x\left(1+rKn\right)+1/2$$

### 2.3. Boundary Condition Considering Kn and Key Parameters

In previous studies, for a slip flow regime with *Kn* ≤ 0.1, although the influence of KL on gas particle velocity distribution already exists due to the KL thickness being thinner, if an appropriate slip boundary condition is given, a macroscopic Navier–Stokes model can be used for numerical simulation. But, for the transition flow regime with 0.1 < *Kn* ≤ 10, the KL thickness is thicker, and the gas flow is mainly dominated by the rarefaction effect. The LBM can be used for numerical simulation, but the corresponding slip boundary condition needs to be reestablished. The commonly used slip boundary condition is the second-order slip boundary condition. The second-order slip boundary condition can be expressed as [17]:

(17) $$u_{s}=A_{1}\lambda_{e}{\left.\frac{\partial u}{\partial n}\right|}_{wall}-A_{2}{\lambda_{e}^{2}\left.\frac{\partial^{2}u}{\partial n^{2}}\right|}_{wall}$$

where **u***_s_* is the slip velocity, **n** is the normal unit vector, *A*_1_ and *A*_2_ are first-order and second-order slip coefficients, and *λ_e_* is the effective molecular mean free path. The effective molecular mean free path obtained from the dynamic viscosity correction function can be expressed as:

(18) $$\lambda_{e}=\mu \varphi (Kn)\sqrt{\pi RT/2}/p$$

Loyalka [40] modified the *A*_1_ using an approximation method in the kinetic theory. The modified *A*_1_ is widely applied in second-order slip boundary conditions [17,31,37]. The modified *A*_1_ can be expressed as:

(19) $$A_{1}=\left(1-0.1817\sigma_{v}\right)\left(2-\sigma_{v}/\sigma_{v}\right)$$

where *σ_v_* is the tangential momentum accommodation coefficient. To compare with other studies’ results, the value of *σ_v_* is consistent with other studies [17,28,41], *σ_v_* = 1.

In Equation (17), when *A*_2_ approaches 0, the second-order slip boundary condition changes to the first-order slip boundary condition, which can be used to predict the velocity distribution in the 0.001 ≤ *Kn* < 0.1. When *A*_2_ approaches a fixed value, it can be used to predict the velocity distribution in the 0.1 ≤ *Kn* < 10. Therefore, *A*_2_ can be constructed by taking different values under different conditions.

Currently, *A*_2_ is generally a constant or a function of the *A*_1_ in Table 1. Using a fixed value can predict the velocity distribution in the 0.1 ≤ *Kn* < 10 reasonably well, but it can produce significant errors in predicting the velocity distribution in the 0.001 ≤ *Kn* < 0.1. This is mainly because in this *Kn* range, relaxation time, dynamic viscosity, and molecular mean free path all vary with *Kn*, but the boundary condition does not reflect the influence of *Kn*. Therefore, the influence of *Kn* should be considered when constructing *A*_2_.

Based on the idea of different values of the *A*_2_ under different conditions and the requirement of considering the change in *Kn* when constructing the second-order slip coefficient, *A*_2_ is a power function of *Kn*:

(20) $$A_{2}=j{Kn}^{h}$$

where *j* and *h* are adjustable parameters. In Section 3 of this work, we will analyze the sensitivity of the numerical results to the adjustable parameters *j* and *h* and ultimately determine the adjustable parameter values. In this work, the second-order slip boundary condition can be expressed as:

(21) $$u_{s}=\left(1-0.1817\sigma_{v}\right)\left(\frac{2-\sigma_{v}}{\sigma_{v}}\right){\left.\frac{\partial u}{\partial n}\right|}_{wall}-j{Kn}^{h}{\lambda_{e}^{2}\left.\frac{\partial^{2}u}{\partial n^{2}}\right|}_{wall}$$

In the LBM, the application of second-order slip boundary conditions requires the use of a discrete treatment scheme, which reflects wall slip. Currently, there are mainly two types of discrete schemes for slip boundary conditions. One is the Discrete Maxwellian scheme (DM) [21] and the other is the combined bounce-back/specular-reflection scheme (CBBSR) [24]. Guo et al. [28] pointed out that these two schemes are identical in a parametric range, and both contain some discrete effects. This work will adopt the CBBSR. The CBBSR can be expressed as:

(22) $$f_{i}\left(x_{w},t\right)=\left(1-r_{b}\right)f_{i}^{SR}\left(x_{w},t\right)+r_{b}f_{i}^{BB}\left(x_{w},t\right)+2r_{b}\rho w_{i}\xi_{i}\cdot u_{w}/\left(∆x/∆t\right)$$

where **x***_w_* is the wall lattice, *f_i_^SR^*(**x***_w_*,*t*) is the distribution function of the specular-reflection particles at the wall lattice, *f_i_^BB^*(**x***_w_*,*t*) is the distribution function of the bounce-back particles at the wall lattice, and *r_b_* is the bounce-back proportion parameter. The parameter *r_b_* can be expressed as [17]:

(23) $$r_{b}=\frac{1}{1+A_{1}\sigma_{v}\sqrt{\pi /6}}$$

## 3. Numerical Results and Analysis

### 3.1. Sensitivity Analysis of Adjustable Parameters

In the second-order slip coefficient Equation (20), there are two adjustable parameters, *j* and *h*. The value of these two adjustable parameters will affect the accuracy of the numerical results in a multi-flow regime. The Taguchi method [49] is used to determine the sensitivity of parameters. Based on the sensitivity analysis results of the Taguchi method, the value of the adjustable parameters can be determined by comparing the dimensionless flow rate with the linearized Boltzmann equation solutions.

Firstly, different combinations of adjustable parameters are established according to the value of the adjustable parameters in Table 2, each called a level. Secondly, monitor points are selected within the range of *Kn* studied in this work and give a dimensionless flow rate for the linearized Boltzmann equation solutions in Table 3. Thirdly, the average difference of the dimensionless flow rate in monitor points between this work and the linearized BE solutions at three levels is calculated, and the range value between the maximum and the minimum average difference for three levels of adjustable parameters is calculated in Table 4 and Table 5; the greater the range value, the greater the sensitivity of the dimensionless flow rate to corresponding adjustable parameter. Finally, according to the sensitivity result, the adjustable parameters that have a greater influence on the dimensionless flow rate are determined first, and then the adjustable parameters that have less influence on the dimensionless flow rate are determined last.

The dimensionless flow rate obtained from the second-order slip boundary conditions in Section 2.3 can be expressed as:

(24) $$Q=\frac{Kn\sqrt{\pi }}{12}+\left(1-0.1817\sigma_{v}\right)\left(\frac{2-\sigma_{v}}{\sigma_{v}}\right)\frac{\sqrt{\pi }}{2}+2j{\left(\frac{\pi }{4}\right)}^{h}{\left(\frac{Kn\sqrt{\pi }}{2}\right)}^{-(1+h)}$$

In this work, the *Kn* is used as the characteristic parameter, while the linear Boltzmann equation solution is given through the inverse *Kn*, the relationship between inverse *Kn* and *Kn* can be expressed as [50]:

(25) $$J=\frac{\sqrt{\pi }}{2Kn}$$

where *J* is the inverse *Kn.*

At all six monitor points, the range value of *j* is greater than *h*, indicating that *Q* is more sensitive to *j* than *h.*

**Figure 2:**
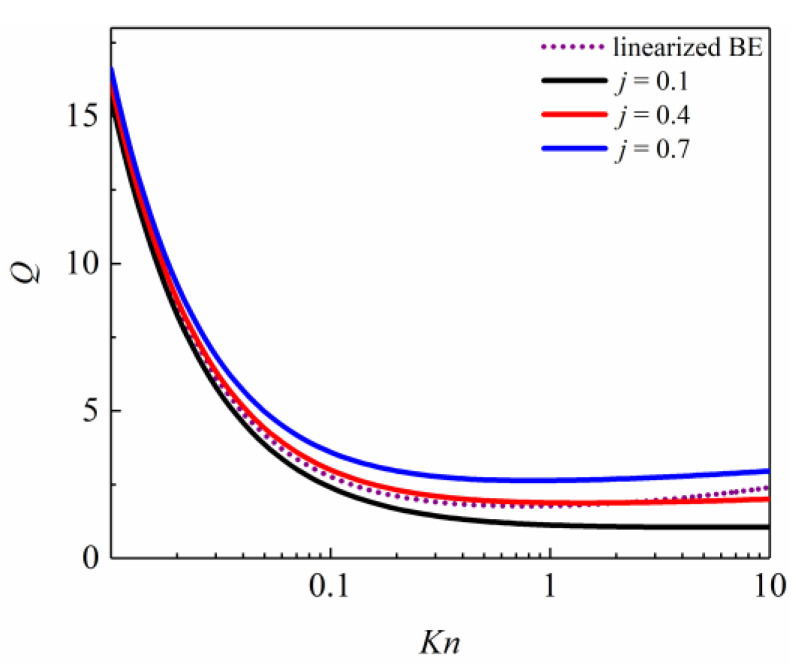
Comparison between the simulated dimensionless flow rate with different *j* and linearized BE solutions by Cercignani et al. [50].

In determining the more sensitive *j*, the less sensitive *h* is tentatively set as a median value of −0.9 at the *h* level in Table 2. Figure 2 shows that when the adjustable parameter *j* is taken as 0.1, 0.4, and 0.7, a comparison of the *Q* of the Poiseuille flow with the linearized BE solutions is made.

In Figure 2, the greater the value of the adjustable parameter *j*, the greater the *Q* of the Poiseuille flow, especially in 0.1 < *Kn* ≤ 10. When the adjustable parameter *j* value is 0.4, the dimensionless flow rate simulated from the second-order slip boundary condition is closest to the linearized BE solutions. Therefore, *j* can be given as 0.4.

**Figure 3:**
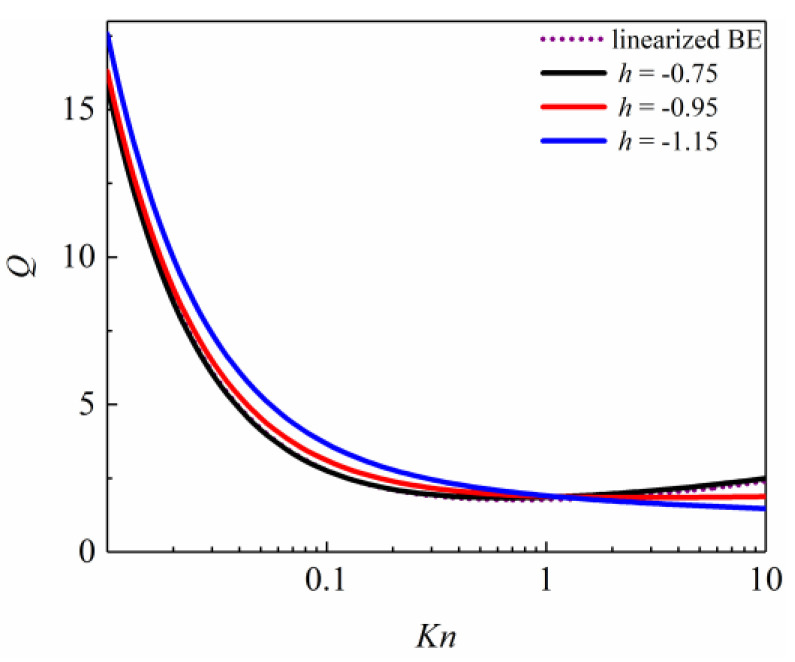
Comparison between the simulated dimensionless flow rate with different *h* and linearized BE solutions by Cercignani et al. [50].

Figure 3 gives *j* as 0.4, and *h* is taken as −0.75, 0.95, and −1.15, and a comparison of the *Q* of the Poiseuille flow with the linearized BE solutions is made.

In Figure 3, when the adjustable parameter *h* changes, the *Q* of the Poiseuille flow shows different variations over different *Kn* numbers; the greater the value of the adjustable parameter *h*, the smaller the *Q* of the Poiseuille flow in 0.001 < *Kn* ≤ 1 and the greater the *Q* of the Poiseuille flow in 1 < *Kn* ≤ 10. When the adjustable parameter *h* value is −0.75, the dimensionless flow rate simulated from the second-order slip boundary condition is closest to the linearized BE solutions. Therefore, the adjustable parameter *h* can be given as −0.75.

**Figure 4:**
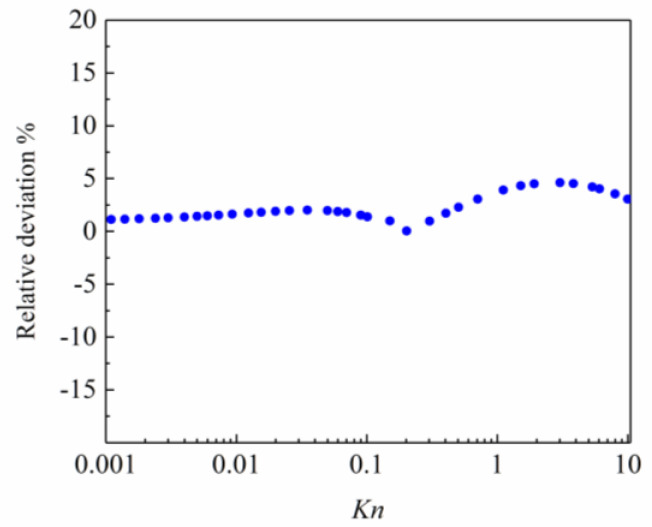
Relative deviation with *Kn*.

Figure 4 gives the variation of the relative deviation of the simulated dimensionless flow rate from the linearized BE solutions with *Kn* when *j* and *h* are taken as 0.4 and −0.75. The maximum relative deviation is within 5%, which indicates that the adjustable parameter values are accurate.

### 3.2. Poiseuille Flow in Microchannel

In this work, 0.001 ≤ *Kn* ≤ 10 includes the continuum flow regime, slip flow regime, and transition flow regime. The following is a comparison and verification analysis of numerical results with the Poiseuille flow in different *Kn*.

**Figure 5:**
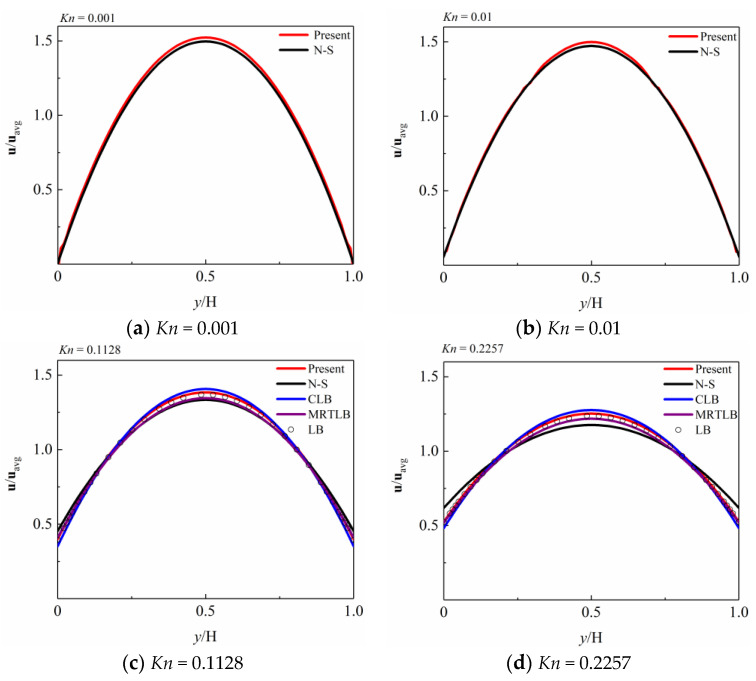

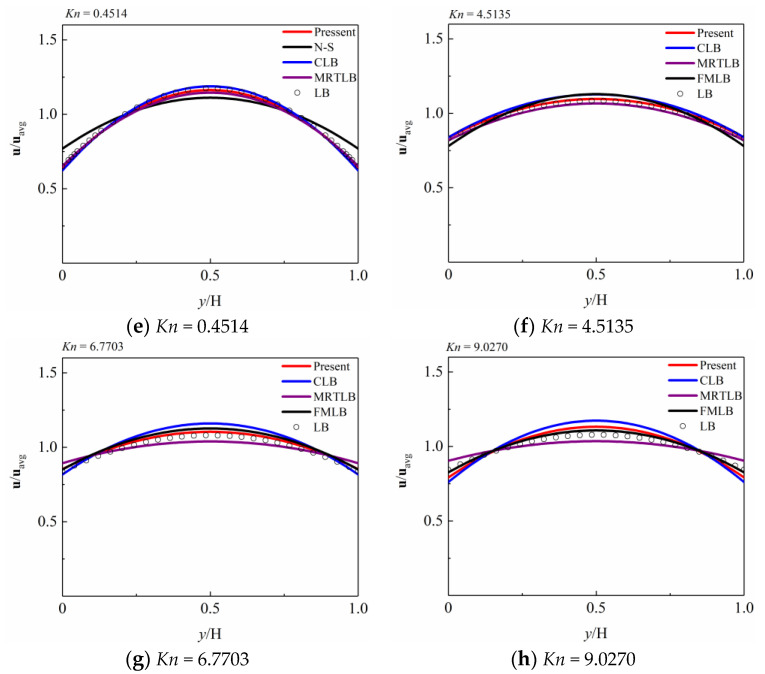
Velocity profile of Poiseuille flow in a microchannel. From figure (**a**–**h**) are velocity profile with different *Kn*. (**a**,**b**) N-S by Bahukudumbi and Beskok [47]. (**c**–**e**) N-S by Hadjiconstantinou [42], CLB by Liu and Feng [17], MRTLB by Guo et al. [28], LB by Ohwada [51]. (**f**–**h**) CLB by Liu and Feng [17], MRTLB by Guo et al. [28], FMLB by Zhou and Zhong [41], LB by Ohwada [51].

In previous studies, different methods were used in different flow regimes. The applicable range of the N-S method is *Kn* < 0.1. In order to compare with other simulation methods, the LB method is often used as a benchmark in 0.1 < *Kn* ≤ 10. In addition, in order to show the advantages of the present numerical simulation method in this work, the results of some methods applicable to different *Kn* are also given in Figure 5.

The conventional Navier–Stokes (N-S) method using a first-order slip boundary condition with *A*_1_ = *Kn*/(1 − *bKn*) [12] and a second-order slip boundary condition with *A*_1_ = 1.11 and *A*_2_ = 0.61 [42], the Central Lattice Boltzmann (CLB) method using *A*_1_ = 0.8183 and *A*_2_ = 0.55 [17], the Multiple Relaxation Times Lattice Boltzmann (MRTLB) method using *A*_1_ = 0.8183 and *A*_2_ = 0.65 [28], the Filter Matrix Lattice Boltzmann (FMLB) method using *A*_1_ = 0.8183 and *A*_2_ = 0.8 [41], and the solution of the linearized Boltzmann (LB) equation [51] are presented in Figure 5 for comparison.

The N-S method is combined with the second-order slip boundary condition, extending the N-S method from the continuum flow regime to the slip flow regime. In the CLB method, the collision process is performed in terms of central moments in ascending order in a moving reference frame, beginning with the lowest and ending with the highest. The MRTLB method has the potential to capture the Knudsen layer by employing the geometry-dependent relaxation time. The FMLB proposes a modified second-order slip boundary condition to give a satisfactory result of the gas flow for the high *Kn* microchannel.

As shown in Figure 5, the non-dimensional velocity is defined by **u**/**u**_avg_, where $u_{\mathrm{avg}}=(1/H)\int_{0}^{H}udy$. *y*/H is the distance of the wall. In Figure 5a,b, the velocity profile simulated by the present numerical simulation method is basically consistent with the N-S method.

As shown in Figure 5c–e, the velocity profile simulated by the present numerical simulation method in this work is very close to those obtained using the CLB and MRTLB, and is more consistent with the LB, which is often used as a benchmark for other methods. In addition, it can be seen in Figure 5c–e that the results obtained using the N-S have significantly deviated from those obtained using other methods, as *Kn* has exceeded the range for the N-S method.

As shown in Figure 5f–h, the velocity profile simulated by the present numerical simulation method in this work is very close to those obtained using the FMLB and LB. In Figure 5g,h, *Kn* exceeded the range of *Kn* (0.1~5) applicable to the CLB and MRTLB, so the simulated results obtained using the CLB and MRTLB have significantly deviated from the FMLB, the LB, and the present numerical simulation method proposed in this work.

**Figure 6:**
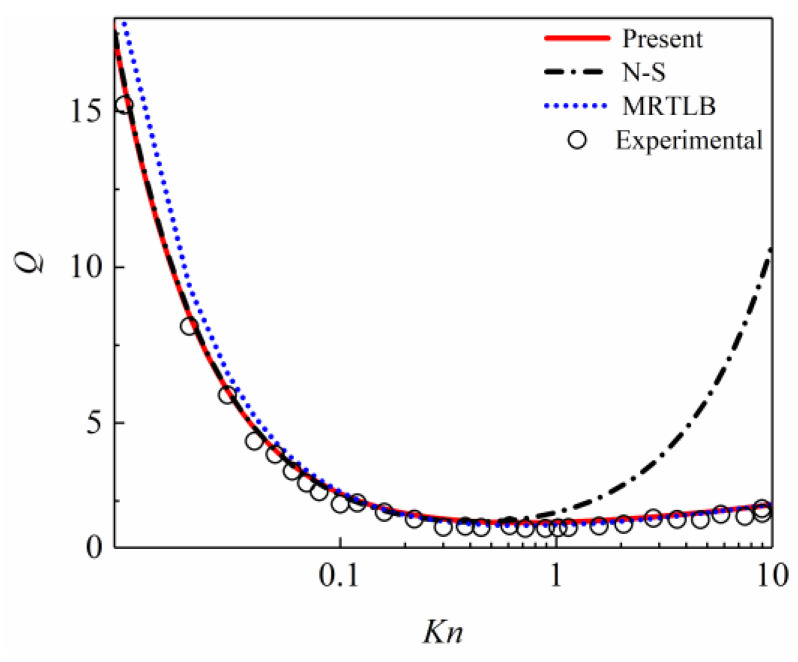
Dimensionless flow rate of Poiseuille flow in a microchannel. Chain line is the N-S method by Hadjiconstantinou [42], dotted line is the MRTLB method by Guo et al. [28], and circle is the Experimental method by Dong [52].

In order to further verify the accuracy of the numerical simulation method in this work and its advantages compared to other models, Figure 6 shows the variation of the dimensionless flow rate in the Poiseuille flow with *Kn* obtained using the present numerical simulation method in this work, as well as the experimental result [52], and the comparison between the N-S [42] method and the MRTLB [28] method. The experimental results are of a rectangular cross-section, and since the long side of the rectangle is much larger than the short side, it can be considered a 2D flow [19,28].

As can be seen in Figure 6, the dimensionless flow rate obtained using the present numerical simulation method in this work agrees with the experimental result in 0.001 ≤ *Kn* ≤ 10, the N-S in *Kn* ≤ 0.3, and the MRTLB in 0.05 ≤ *Kn* ≤ 10. In Figure 6, it can also be seen that when *Kn* is less than 0.3, the dimensionless flow rate obtained using the N-S is in good agreement with the experimental result, but when *Kn* is greater than 0.3, the dimensionless flow rate obtained using the N-S significantly deviates from the experimental result, the MRTLB method, and the present method. The dimensionless flow rate obtained using the MRTLB is in good agreement with the experimental results in 0.05 ≤ *Kn* ≤ 10; however, when *Kn* is less than 0.05, there will be a deviation between the dimensionless flow rate obtained using the MRTLB, the N-S, the present method, and the experimental results. The second-order slip boundary condition adopted by the MRTLB and is the second-order slip coefficient of a fixed value, and the slip velocity distribution obtained by MRTLB would be higher than the experiment in *Kn* < 0.05, Therefore, there is a deviation in the experimental results of the dimensionless flow rate comparison.

### 3.3. Pulsating Flow in a Microchannel

At present, some studies show that pulsating flow in microchannels can enhance heat transfer [53,54,55]. The results of pulsating flow showed that forced pulsations could enhance average heat transfer. The enhancement of heat transfer depends on the flow patterns. The amplitude and frequency of pulsations could produce different flow phenomena, which ultimately affect the effect of heat transfer. However, there are currently few or no studies on the pulsating flow in a microchannel with a multi-flow regime. The pulsating flow patterns are still unclear with the multi-flow regime. In order to verify that the present numerical simulation method in this work can be used for the pulsatile flow in the microchannel, the following is a comparison and verification analysis of numerical results with experimental results [56].

One of the dimensionless numbers commonly used in pulsating flow is the Womersley number. It was first stated in the research of biomechanics and pulsating blood flow. It represents the relationship between the frequency of pulsating flow and the viscous effect. The Womersley number, which is expressed by *α* (or Wo), is obtained as follows [53]:

(26) $$\alpha =\frac{H}{2}\sqrt{\frac{\omega }{\nu }}$$

where *ω* is the angular frequency of the vibration and *ν* is the kinematic viscosity. When the *α* is greater than 1, at certain specific phases of the pulsating flow, the velocity near the microchannel wall area will be greater than the velocity in the channel center, and the velocity profile of the microchannel will be annular-shaped. This annular-shaped velocity profile of the pulsating flow is called the annular effect [57,58].

**Figure 7:**
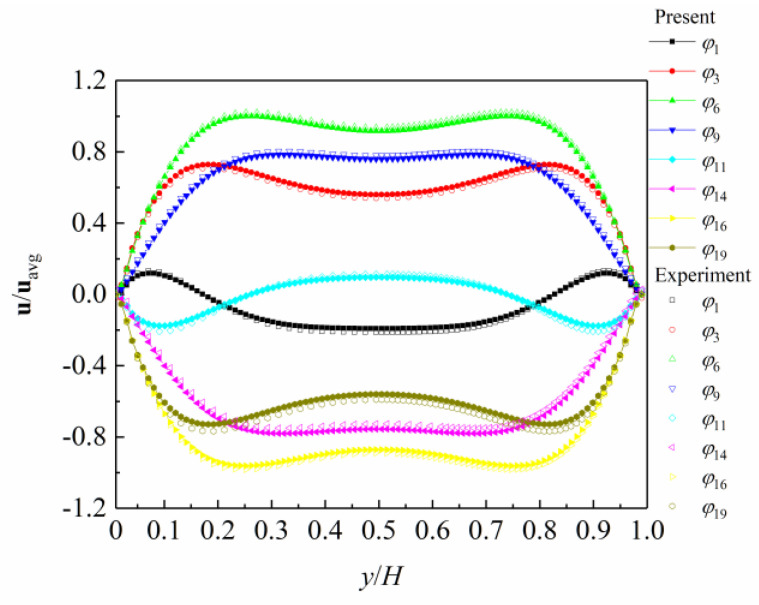
Velocity profile of pulsating flow in a microchannel.

Shi and Jaworski [56] used the Planar Laser Induced Fluorescence (PLIF) and Particle Image Velocimetry (PIV) methods to measure the velocity profile of sinusoidal pulsating flow in a microchannel, obtaining the velocity profile at 20 phases. Figure 7 shows a comparison between the velocity profile of different phases obtained by the model in this work and the experimental results when the microchannel inlet velocity is a sinusoidal pulsating flow with *a* = 8. The geometric parameters and gas parameters of the pulsating flow are consistent with the experiment work.

As shown in Figure 7, the velocity profile of different phases obtained by numerical simulation is in good agreement with the experimental result, and the phenomenon of the annular velocity profile in the microchannel is reflected in the phases of *φ*_1_ and *φ*_11_, as well as in the phases of *φ*_6_ and *φ*_16_, which accurately simulate the annular effect of pulsating flow in the microchannel.

## 4. Conclusions

This work used a numerical simulation method for gas flow based on the lattice Boltzmann equation, which is applicable to the multi-flow regime. The results are summarized as follows.

1. The relationship between the modified relaxation time and *Kn* was determined. The Bosanquet-type correction function is employed to capture the rarefaction effects, and the values of the rarefaction factor in the correction function were determined to be 2 (0.001 < *Kn* ≤ 0.3), 1.7 (0.3 < *Kn* ≤ 2), and 1.5 (2 < *Kn* ≤ 10), respectively;
2. The relaxation time, the dynamic viscosity, and the molecular mean free path all vary with *Kn*. In this work, we innovatively introduce the *Kn* number into the second-order slip boundary condition, as a power function is established, and the CBBSR boundary scheme is employed, which can obtain multi-flow regime results in microchannels;
3. The sensitivity analysis of the adjustable parameters of the second-order slip coefficient was carried out, and the results show that *Q* is more sensitive to *j* than *h.* The values of the adjustable parameters were determined to be *j* = 0.4 and *h* = −0.75;
4. The numerical simulation method was validated from the aspects of the Poiseuille flow velocity profile and dimensionless flow rate in the microchannel, as well as the annular effect of the pulsating flow velocity profile in the microchannel. The results show the velocity profile and the dimensionless flow rate simulated by the present numerical simulation method in this work, and the phenomenon of the annular velocity profile in the microchannel is reflected in the phases of *φ*_1_ and *φ*_11_, as well as in the phases of *φ*_6_ and *φ*_16_;
5. For potential future research, the present numerical simulation method can also be used for the pulsating flow in the multi-flow regime. For pulsating flow, the effect of wall slip velocity on the annular effect, as well as the difference between mesoscopic and macroscopic, can be further investigated.

## Figures and Tables

**Table 1:** Values of slip coefficients proposed in the literature.

Source	*A*_1_	*A*_2_
Hadjiconstantinou [42]	1.11	0.61
Maxwell [11]	(2 − *σ_v_*)/*σ_v_*	0
Beskok [13]	[(2 − *σ_v_*)/*σ_v_*]·[*Kn*/(1 − *bKn*)]	0
Hisa [43]	(2 − *σ_v_*)/*σ_v_*	0.5
Aubert [44]	(2 − *σ_v_*)/*σ_v_*	9/8
Schamberg [45]	(2 − *σ_v_*)/*σ_v_*	5π/12
Zhang [46]	1.1466	0.31
Bahukudumbi [47]	1.29777 + 0.71851tan^−1^(−1.17488*Kn*^0.58642^)	0
Liu [17]	(1 − 0.1817*σ_v_*) (2 − *σ_v_*)/*σ_v_*)	0.55
Li [37]	(1 − 0.1817*σ_v_*) (2 − *σ_v_*)/*σ_v_*)	0.8
Guo [28]	(1 − 0.1817*σ_v_*) (2 − *σ_v_*)/*σ_v_*)	1/π + (*A*_1_)^2^/2
Deissler [48]	(2 − *σ_v_*)/*σ_v_*	1.6875

**Table 2:** The level of the adjustable parameter.

Level	*j*	*h*
1	0.1	−0.7
2	0.5	−0.9
3	0.9	−1.1

**Table 3:** Correspondence between *J* and *Kn* at different monitor points with *Q*.

Monitor Points	P_1_	P_2_	P_3_	P_4_	P_5_	P_6_
*J*	100	20	3	1	0.3	0.1
*Kn*	0.008	0.044	0.295	0.886	2.954	8.862
*Q*	17.689	4.394	1.710	1.539	1.702	2.033

**Table 4:** Average difference and range under P_1_, P_2_, and P_3_.

Adjustable Parameter	Level	Average Difference			Range Value		
		P_1_	P_2_	P_3_	*R* (P_1_)	*R* (P_2_)	*R* (P_3_)
*j*	0.1	−1.7094	0.1011	0.2561	1.6817	1.6869	1.8241
	0.5	−2.5503	−0.7424	−0.6561			
	0.9	−3.3911	−1.5858	−1.568			
*h*	−0.7	−1.7877	−0.1691	−0.3673	1.5001	1.2802	0.6046
	−0.9	−2.2755	−0.6085	−0.6293			
	−1.1	−3.2878	−1.4493	−0.9719			

**Table 5:** Average difference and range under P_4_, P_5_, and P_6_.

Adjustable Parameter	Level	Average Difference			Range Value		
		P_4_	P_5_	P_6_	*R* (P_4_)	*R* (P_5_)	*R* (P_6_)
*j*	0.1	0.3983	0.6429	0.9602	1.990	2.2707	2.6473
	0.5	−0.5966	−0.4924	−0.3633			
	0.9	−1.5917	−1.6278	−1.6871			
*h*	−0.7	−0.5371	−0.7726	−1.0717	0.1202	0.5430	1.3268
	−0.9	−0.5957	−0.4751	−0.2735			
	−1.1	−0.6573	−0.2296	0.2551			

## Data Availability

Data are contained within the article.
